# Vitamin C in Cultured Human (HeLa) Cells: Lack of Effect on DNA Protection and Repair

**DOI:** 10.3390/nu5041200

**Published:** 2013-04-09

**Authors:** Amaya Azqueta, Solange Costa, Yolanda Lorenzo, Nasser E. Bastani, Andrew R. Collins

**Affiliations:** 1 Department of Pharmacology and Toxicology, University of Navarra, C/Irunlarrea 1, 31009 Pamplona, Spain; 2 Department of Nutrition, Institute of Basic Medical Sciences, University of Oslo, PB 1046 Blindern, 0316 Oslo, Norway; E-Mails: solange.costa2@gmail.com (S.C.); y.c.lorenzo@medisin.uio.no (Y.L.); n.e.bastani@medisin.uio.no (N.E.B.); a.r.collins@medisin.uio.no (A.R.C.); 3 Environmental Health Department, National Institute of Health, Rua Alexandre Herculano 321, 4000-055 Porto, Portugal

**Keywords:** DNA damage, DNA protection, DNA repair, vitamin C

## Abstract

Aims: Dietary antioxidants, including vitamin C, may be in part responsible for the cancer-preventive effects of fruits and vegetables. Human intervention trials with clinical endpoints have failed to confirm their protective effects, and mechanistic studies have given inconsistent results. Our aim was to investigate antioxidant/ pro-oxidant effects of vitamin C at the cellular level. Experimental approach: We have used the comet assay to investigate effects of vitamin C on DNA damage, antioxidant status, and DNA repair, in HeLa (human tumor) cells, and HPLC to measure uptake of vitamin C into cells. Results: Even at concentrations in the medium as high as 200 μM, vitamin C did not increase the background level of strand breaks or of oxidized purines in nuclear DNA. Vitamin C is taken up by HeLa cells and accumulates to mM levels. Preincubation of cells with vitamin C did not render them resistant to strand breakage induced by H_2_O_2_ or to purine oxidation by photosensitizer plus light. Vitamin C had no effect on the rate of repair of strand breaks or oxidized bases by HeLa cells. However, vitamin C at a concentration of less than 1 μM, or extract from cells preincubated for 6 h with vitamin C, was able to induce damage (strand breaks) in lysed, histone-depleted nuclei (nucleoids). Conclusion: In these cultured human cells, vitamin C displays neither antioxidant nor pro-oxidant properties; nor does it affect DNA strand break or base excision repair.

## 1. Introduction

Vitamin C (ascorbic acid) is one of the best known and most studied dietary antioxidants. Its antioxidant credentials are well-established *in vitro*, since it can readily be demonstrated to prevent oxidation of lipids, DNA and other biological molecules. However, it is also possible for vitamin C to act as a pro-oxidant, through its ability to reduce transition metal ions, thus promoting the Fenton reaction which, acting on peroxides, produces highly reactive hydroxyl radicals [1]. The effect of vitamin C *in vivo* has been investigated in human trials with DNA oxidation as the measured endpoint. They include single dose intervention and longer trials, typically of a few weeks with daily supplementation. The first single-dose study was carried out by Green *et al.* [2], who administered 35 mg/kg of vitamin C to six healthy volunteers after an overnight fast and found a protection against γ radiation-induced DNA breaks in white blood cells, using the comet assay (described below). Since then, more than 20 intervention studies with vitamin C have been carried out, and they have been critically reviewed by Duarte and Lunec [3] who find that most of the studies show either a decrease in DNA oxidation or no effect, while there are some that show an increase in DNA lesions. Møller and Loft [4] also describe conflicting results with vitamin C, concluding that more studies with better designs to avoid bias should be made in order to understand the role of vitamin C in protecting against DNA oxidation. In a subsequent supplementation trial, Møller *et al.* [5] found that a slow release formulation of vitamin C had a more pronounced and sustained protective effect on the steady state level of DNA base oxidation than did a normal release formulation. 

Herbert *et al.* [6] carried out a double-blind placebo-controlled trial giving different doses of vitamin C (0, 80, 200 and 400 mg/day) to four groups of 40 healthy volunteers over a period of 15 weeks (plus a 10 week washout period). They showed that vitamin C did not affect the intracellular level of 8-oxodGuo measured by HPLC. (This challenged an earlier report that vitamin C caused oxidation of bases in DNA [7]). 

Sram *et al.* [8] recently reviewed human studies based on measurement of a variety of biomarkers of genetic damage—including some trials aimed at specific groups with occupational/environmental exposure to genotoxins. Generally either a protective effect or no effect was seen, depending—they suggest—on factors such as individual diet-derived vitamin C concentrations, levels of exposure to xenobiotics, and oxidative stress.

Bjelakovic *et al*. [9] published a meta-analysis of randomized trials of antioxidant supplements aimed at primary and secondary prevention of various diseases, with mortality as the endpoint. They concluded that antioxidant supplements have no beneficial effects on mortality, though in the case of vitamin C the number of individuals sampled is still relatively small and a definitive statement cannot be made. 

Reviews of this subject conclude that we still need more studies to clarify the effect of vitamin C in humans [1,8,9,10]. We considered that it would be useful to investigate effects of vitamin C on genetic stability in cell culture, and so have examined, in the human tumor cell line HeLa, potential pro-oxidant, DNA-damaging effects of the vitamin; protection against DNA breakage caused by H_2_O_2_; and protection against base oxidation induced by photosensitizer plus visible light. In addition, we investigate the possibility that vitamin C might influence the capacity of cells for DNA repair.

We applied the comet assay, as used in many human biomonitoring studies, to the measurement of DNA damage, both strand breaks (SBs) and oxidized bases (employing the enzyme formamidopyrimidine DNA glycosylase (FPG) which converts 8-oxoGua and breakdown products of damaged purines to SBs). We used two approaches to measure repair of DNA damage [11]. If cells are subjected to damage (SBs or base oxidation), and then incubated to allow cellular repair, the residual lesions can be measured at intervals to show the kinetics of damage removal. Alternatively, in an *in vitro* assay, a cell extract is incubated with substrate DNA containing specific damage. The ability of the extract to introduce DNA breaks in the substrate cells reflects the activity of the enzymes responsible for the initial steps of repair, *i.e.*, removing the lesion. 

## 2. Materials and Methods

### 2.1. Cell Culture

HeLa cells (derived from human cervical cancer) were grown in DMEM supplemented with 10% fetal bovine serum and antibiotics (100 U/mL penicillin and 100 μg/mL streptomycin). According to the formulation for DMEM it does not contain vitamin C. Vitamin C was not detectable in the batch of serum used. Cells were maintained as monolayer cultures at 37 °C in a humidified atmosphere with 5% CO_2_ and routinely passaged by trypsinization when nearly confluent.

### 2.2. Cell Treatment for DNA Damage and Protection

HeLa cells in culture medium were incubated with 0, 5, 25, 50, 100 or 200 µM vitamin C (ascorbic acid) for 30 min or 6 h (3 h incubation, followed by a wash with PBS and a further 3 h incubation with vitamin C at the same concentration) at 37 °C in the dark. The two consecutive 3 h treatments were designed to allow for the possible instability of vitamin C in solution. Vitamin C was dissolved in PBS in the dark just before use in each experiment. After treatment the comet assay was performed as described below. To check for DNA protection, after vitamin C treatment, cells were washed with PBS and then treated on ice with 25 µM H_2_O_2_ for 5 min to induce SBs, or with 1 µM of the photosensitizer Ro (Ro-19-8022, from F. Hoffmann-La Roche) plus 1.5 min visible light (500 W tungsten halogen lamp, at 33 cm on ice) to induce oxidized purines, mostly 8-oxoGua. After treatment the comet assay was performed (see below). Three independent experiments were carried out.

### 2.3. Measuring Uptake of Vitamin C

Near-confluent cultures of HeLa cells in 60 mm dishes were incubated for 6 h (3 h + wash + 3 h, as above) with vitamin C at 50 or 200 µM. At the end of this incubation, or after a further 24 h incubation in fresh medium without vitamin C, cells were washed with PBS and scraped with a silicone rubber scraper into suspension in PBS. A cell count was carried out. The suspension was centrifuged (400× *g*, 5 min) and the pellet resuspended in 250 µL of PBS to which an equal volume of 10% metaphosphoric acid was added before storing at −20 °C. 

The frozen, acidified samples were thawed, and centrifuged (3500× *g* at 4 °C for 10 min). A volume of 100 μL clear supernatant was mixed with 400 μL of the mobile phase (2% acetonitrile in 2.5 mM NaH_2_PO_4_, 2.5 mM dodecyltrimethyl ammonium chloride and 1.25 mM Na_2_EDTA in Milli-Q water) for direct determination of vitamin C by high-performance liquid chromatography (HPLC). For separation of vitamin C from interfering sample constituents, a Chromolith Performance RP18-e, 4.6 mm × 100 mm column was used, with a Chromolith Performance RP18-e, 4.6 mm × 10 mm guard column (Phenomenex, Torrance, USA). The injection volume was 5 μL and the flow rate was 6.0 mL/min. A variable wavelength ultraviolet (UV) detector was used at 264 nm.

### 2.4. Treatment of Cells for Cellular Repair Assay

Repair of DNA damage can be studied by treating cells with a DNA-damaging agent, incubating, and measuring the damage remaining at intervals. Using this cellular repair assay we investigated the kinetics of DNA SB rejoining and base excision repair (BER). To check the influence of vitamin C on the repair of SBs, HeLa cells were pre-incubated with 0, 50 or 100 µM vitamin C overnight. Then cells were washed with PBS, treated with 0 or 100 µM H_2_O_2_ for 5 min on ice, and incubated for 10, 30 or 60 min in culture medium. To check the influence of vitamin C on the repair of oxidized bases, pre-incubation overnight with 0, 50 or 100 µM vitamin C was followed by a wash with PBS, treatment with 0 or 1 µM Ro plus 5 min of light, and incubation for 1, 2, 4, 6, 8, or 24 h in culture medium. In another experimental design, HeLa cells in culture medium were pre-incubated with a higher concentration (200 µM) of vitamin C but for only 30 min. Then cells were washed with PBS, treated with 1 µM Ro plus 2.5 min of light, and incubated for 3 h in culture medium including 0 or 200 µM vitamin C. They were then washed with PBS and incubated again in culture medium with the same concentration of vitamin C for a further 3 h. The comet assay was performed after each time of incubation. The concentration of vitamin C used in these experiments was not genotoxic. Three independent experiments were performed.

### 2.5. Comet Assay

Just after treatment (or after incubation for repair), HeLa cells were trypsinized and resupended in PBS (1 × 10^6^ cells/mL). Thirty μL of each cell suspension were mixed with 140 μL of 1% low melting point agarose, and two drops of 70 μL were spread onto microscope slides precoated with 1% of normal melting point agarose. Glass cover slips were placed on the drops of agarose, which were allowed to set at 4 °C. Then the cover slips were removed and the cells embedded in agarose were lysed for 1 h by immersion in 2.5 M NaCl, 0.1 M Na_2_EDTA, 0.1 M Tris base, pH 10 and 1% Triton X-100 at 4 °C (lysis solution). For measurement of SBs, the slides were then placed in a horizontal gel electrophoresis tank and the DNA was allowed to unwind for 40 min in freshly prepared alkaline electrophoresis solution (0.3 M NaOH and 1 mM Na_2_EDTA, pH > 13). 

For measurement of oxidized purines, after lysis, slides were washed three times (5 min each time) with buffer F (0.1 M KCl, 0.5 mM Na_2_EDTA, 40 mM HEPES, 0.2 mg/mL BSA, pH 8.0) and incubated for 30 min at 37 °C with FPG in buffer F, or with buffer F alone, in a moist box. After incubation the slides were placed in the electrophoresis solution.

Electrophoresis was carried out in the alkaline solution for 30 min at 1.1 V/cm and approximately 300 mA at 4 °C. The slides were washed in 0.4 M Tris base (pH 7.5) for 10 min at 4 °C to neutralize the excess alkali and 10 min in water at 4 °C. Then they were left to dry overnight. 

Gels were stained with 25 µL of DAPI (4′,6-diamidine-2-phenylindol dihydrochloride, 1 µg/mL), covered with a cover slip and coded before microscopic analysis. DAPI-stained nuclei were evaluated with a Nikon Eclipse TS-100 fluorescence microscope. A total of 50 comets on each gel were visually scored as belonging to one of five classes according to the tail intensity. Each comet class was given a value between 0 and 4: (0) = undamaged and (4) = maximum damage [12]. The total score in arbitrary units (AU) was calculated by the following equation:

(percentage of cells in class 0 × 0) + (percentage of cells in class 1 × 1) + (percentage of cells in class 2 × 2) + (percentage of cells in class 3 × 3) + (percentage of cells in class 4 × 4).

Consequently, the total score was in the range from 0 to 400 arbitrary units. Net H_2_O_2_-induced damage was calculated by subtracting the comet score for non-H_2_O_2_-treated cells from the score + H_2_O_2_. Net FPG-sensitive sites were calculated by subtracting the score for the slide incubated with buffer from the score for the slide incubated with enzyme. Net Ro-induced damage was calculated as the difference in net FPG-sensitive sites between Ro-treated cells and cells not treated with Ro.

### 2.6. *In Vitro* Assay for DNA Repair

The *in vitro* repair assay is used to check the ability of cell extracts to carry out the first steps of BER. Gel-embedded nucleoids from HeLa cells carrying oxidized bases (substrate) were incubated with extracts from HeLa cells pre-incubated with/without vitamin C and with different concentrations of vitamin C directly.

#### 2.6.1. Preparation of Substrate Cells

HeLa cells were grown to 80% confluence, washed with PBS and treated with 1 µM Ro plus 4 min of light. Then they were washed with cold PBS, detached by trypsinization, centrifuged for 5 min at 800× *g* at 4 °C and resuspended in freezing medium (DMEM supplemented with 20% fetal bovine serum, and 10% DMSO) at a concentration of 1 × 10^6^ cells per mL. Aliquots of 0.7 mL were frozen slowly and stored at −80 °C.

#### 2.6.2. Preparation of Cell Extracts

Extracts were prepared from HeLa cells incubated with 0, 50, 100 or 200 µM vitamin C for 6 h (3 h + wash + 3 h). They were washed with cold PBS, trypsinized, centrifuged at 800× *g* for 5 min at 4 °C and resuspended in cold PBS at a concentration of 1.25 × 10^6^ cells per mL. Then the cell suspension was split into aliquots of 1 mL and centrifuged at 14,000× *g* for 5 min at 4 °C. The supernatant of each aliquot was completely discarded and the dry pellets were frozen in liquid nitrogen and stored at −80 °C. After thawing the pellets were resuspended in 32 µL of extraction buffer A (45 mM HEPES, 0.4 M KCl, 1 mM EDTA, 0.1 mM dithiothreitol and 10% glycerol, pH 7.8) containing 0.25% of Triton X-100, mixed using vortex at high speed for 5–10 s, incubated for 5 min on ice and centrifuged at 14,000× *g* for 5 min at 4 °C. Then 27 µL of the supernatant was mixed with 110 µL of cold reaction buffer F.

#### 2.6.3. The Reaction

The substrate cells were thawed, diluted in cold PBS, centrifuged at 800× *g* for 5 min at 4 °C and resuspended in PBS at a concentration of 1 × 10^6^ cells per mL. As described in section 2.4 gels were made, put in lysis for 1 h and washed 3 times (5 min each time) with buffer F. Then 30 µL of extract (experimental treatment), FPG (positive control), or buffer F (negative control) were placed on each gel (two gels per condition) and covered with a cover slip. Slides were placed in a moist box and incubated for 10 and 20 min. Afterwards the rest of the comet assay was performed as described above.

#### 2.6.4. Treatment of Nucleoids with Vitamin C

In order to check the direct effect of vitamin C on DNA, non-damaged HeLa cell nucleoids, or substrate nucleoids prepared as described in Section 2.6.1, were treated for 10 or 20 min with vitamin C dissolved in buffer A at 0, 1, 5 or 25 µM and diluted with buffer F, as described in Section 2.6.2. The reaction was carried out as described in Section 2.6.3. The final concentrations of vitamin C were therefore 0, 0.25, 1.25, and 6.25 μM.

### 2.7. Statistical Analysis

The statistical analysis was performed by using the software SPSS 11.0. Data are presented with descriptive analysis (mean ± SD for 3 independent experiments). The comparisons between total comet scores of the different groups were performed by the non-parametric Kruskal-Wallis test followed by Mann-Whitney U test when the first one showed the presence of differences. Probability *p* ≤ 0.05 was accepted as the level of significance.

## 3. Results

### 3.1. Direct Induction of DNA Damage

**Figure 1:**
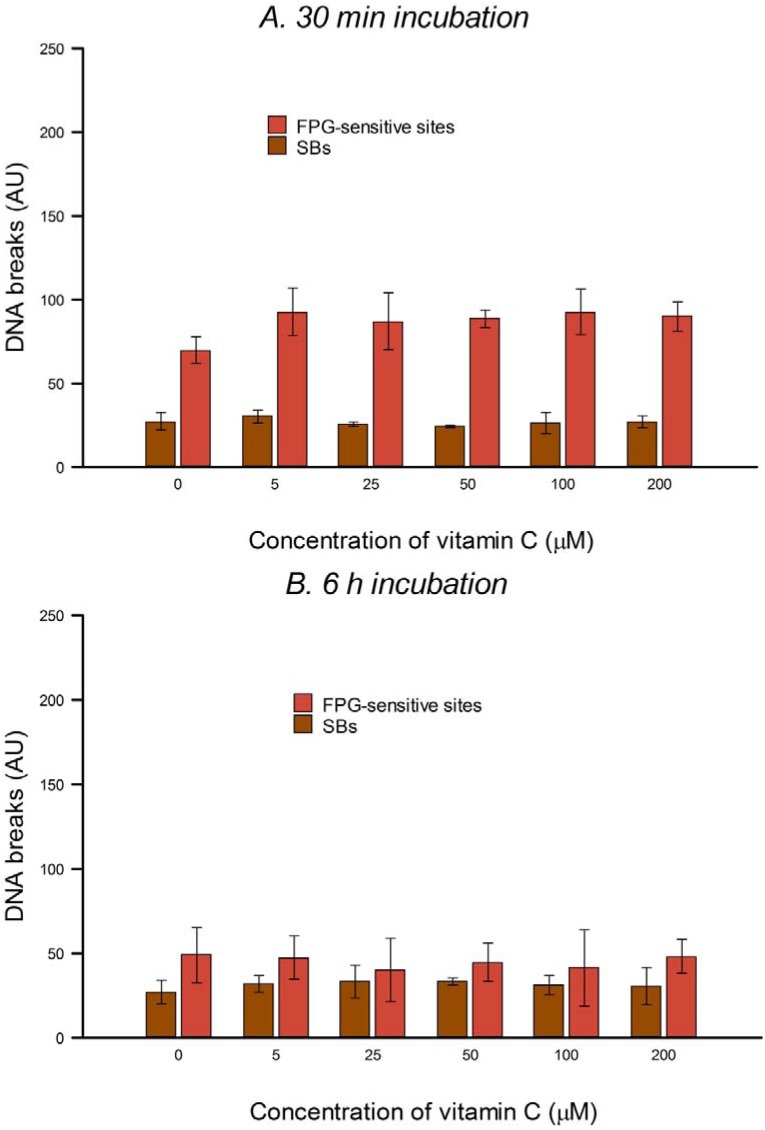
Effect of different concentrations of vitamin C on DNA integrity (SBs and net formamidopyrimidine DNA glycosylase (FPG)-sensitive sites) of HeLa cells treated for 30 min (**A**) and 6 h (**B**) and measured using the comet assay. Bars: SD calculated from the results of 3 independent experiments. No significant effects were seen.

We evaluated DNA damage, *i.e.*, SBs and oxidized bases, just after incubating HeLa cells with different concentrations of vitamin C for 30 min or 6 h, using the comet assay with and without FPG. Treatment with different concentrations of vitamin C (5–200 µM) did not cause or decrease SBs or base oxidation in HeLa cells (Figure 1A,B). 

### 3.2. Protection against H_2_O_2_- and Ro-Induced DNA Damage

**Figure 2:**
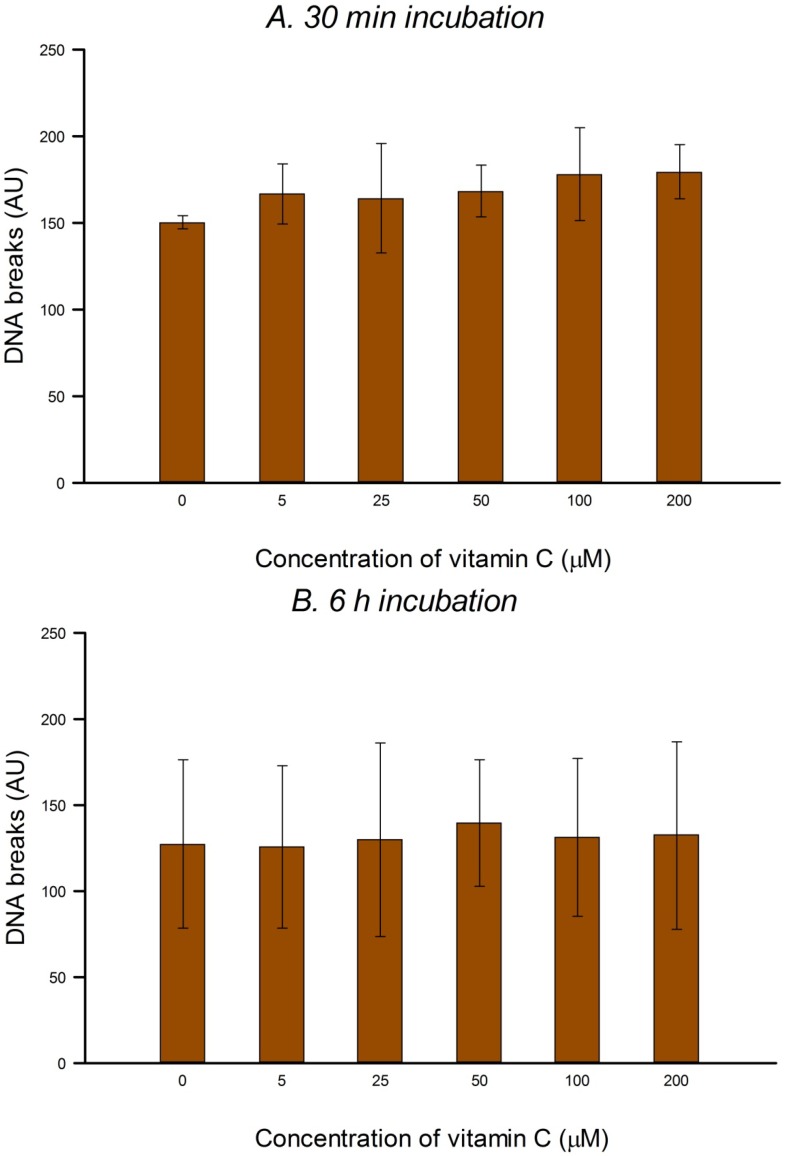
Effect of pre-incubation of HeLa cells with different concentrations of vitamin C for 30 min (**A**) or 6 h (**B**) on damage induced by H_2_O_2_. Net H_2_O_2_-induced damage was calculated by subtracting comet score without H_2_O_2_ (as in Figure 1) from the total comet score with H_2_O_2_. Bars: SD calculated from the results of 3 independent experiments. No significant effects were seen.

**Figure 3:**
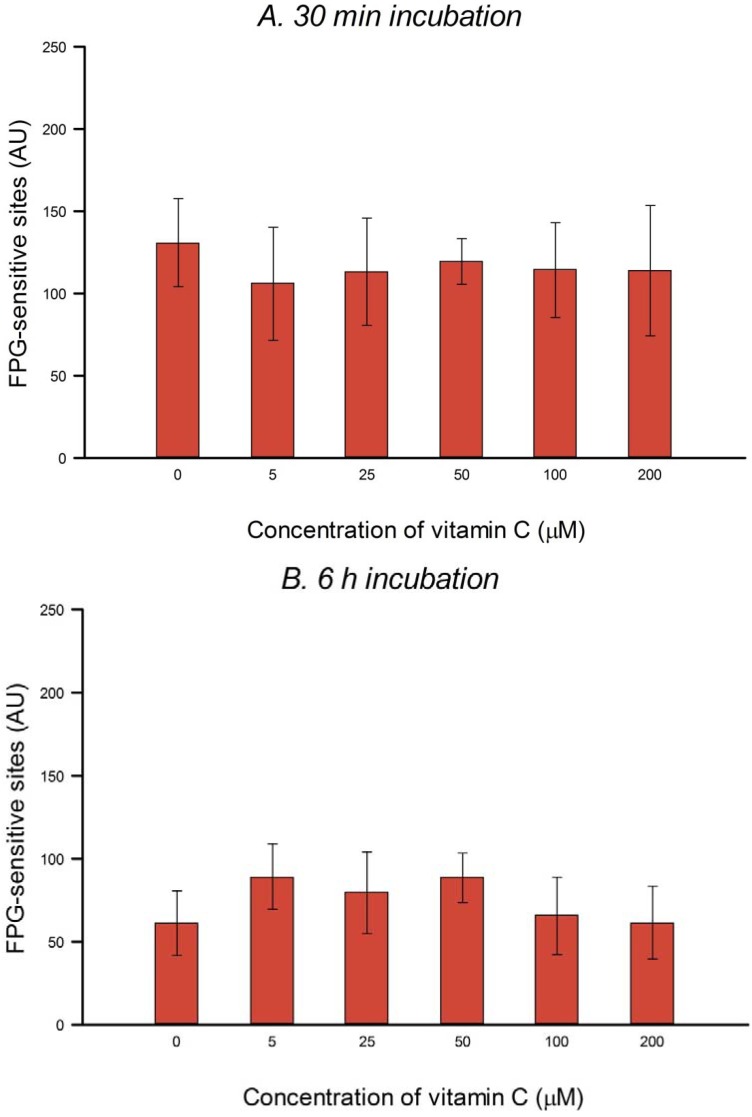
Effect of pre-incubation of HeLa cells with different concentrations of vitamin C for 30 min (**A**) or 6 h (**B**) on FPG-sensitive sites induced by Ro plus light. Net Ro-induced damage was calculated by subtracting the FPG-sensitive sites without Ro plus light (as in Figure 1) from the FPG-sensitive sites with Ro plus light. Bars: SD calculated from the results of 3 independent experiments. No significant effects were seen.

Using the comet assay we investigated the possible protective effect of vitamin C against DNA damage caused by H_2_O_2_ or Ro plus light after pre-incubating HeLa cells with vitamin C (5–200 µM) for 30 min or 6 h. In HeLa cells the yield of damage, net H_2_O_2_-induced breaks or net Ro-induced FPG-sensitive sites, was unchanged by the vitamin C incubation (Figure 2A,B and Figure 3A,B). 

### 3.3. Cellular Repair of SBs

**Figure 4:**
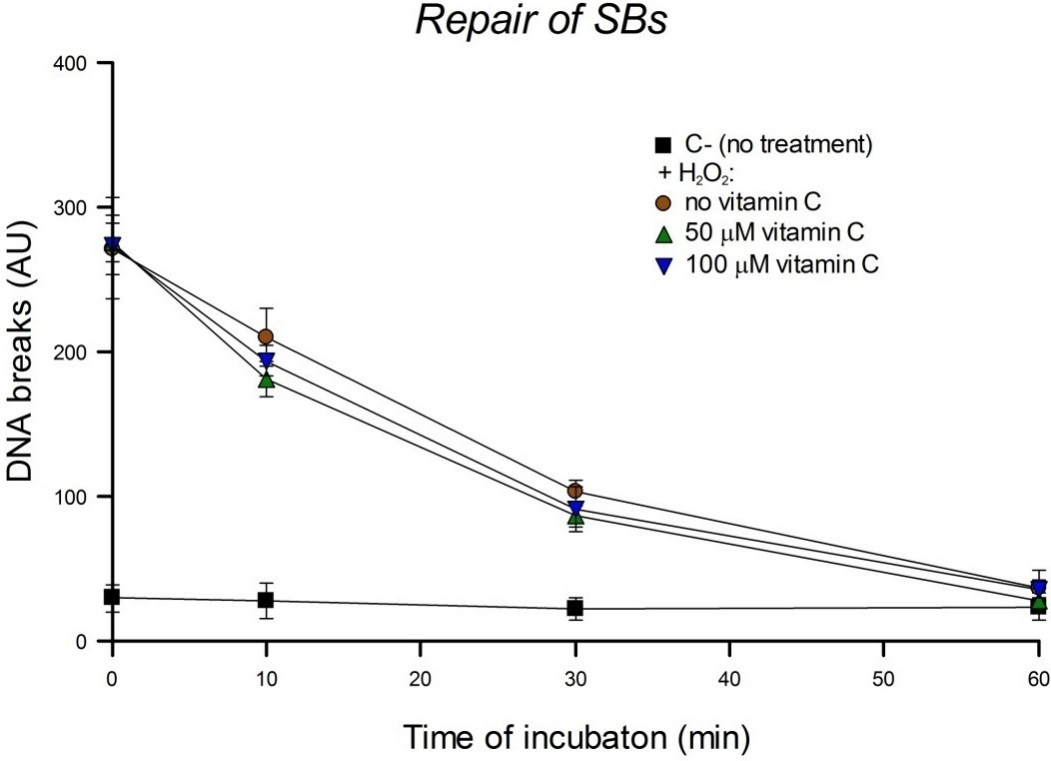
Effect of overnight pre-incubation of HeLa cells with different concentrations of vitamin C on the kinetics of repair of SBs: residual SBs were measured after different times of incubation after H_2_O_2_ treatment. C-: control cells without any kind of treatment. Bars: SD calculated from the results of 3 independent experiments. No significant effects were seen.

We studied the effect of vitamin C on the kinetics of repair of SBs induced by H_2_O_2_ after pre-incubating HeLa cells with different concentrations of vitamin C overnight. After H_2_O_2_ treatment cells were incubated and SBs remaining were measured at intervals. As is seen in Figure 4, rejoining of SBs is rapid, with a t_1/2_ of about 20 min and complete repair by 60 min. Pre-incubation of cells with 50 or 100 µM vitamin C overnight did not affect the repair of the SBs induced by H_2_O_2_.

### 3.4. Cellular Repair of Oxidized Purines

**Figure 5:**
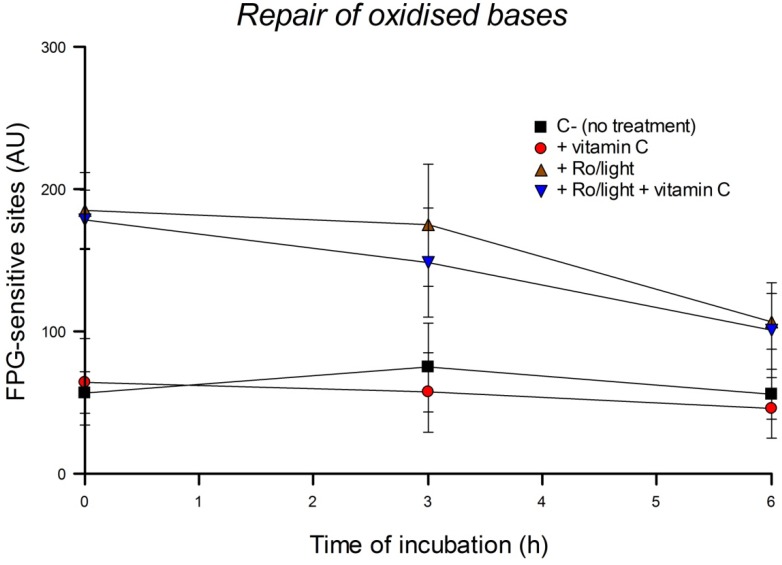
Effect of incubation of HeLa cells with 200 µM of vitamin C before and during the repair of oxidized bases induced by Ro plus light; residual FPG-sensitive sites were measured at different time points. Bars: SD calculated from the results of 3 independent experiments. No significant effects were seen.

We next investigated the effect of vitamin C on the kinetics of BER after pre-incubating HeLa cells with different concentrations of vitamin C overnight. We induced oxidized purines, mostly 8-oxoGua, using Ro plus light. The comet assay was performed after different times of incubation of the treated cells up to 6 h. The pre-incubation of cells with 50 or 100 µM vitamin C overnight did not affect the removal of the FPG-sensitive sites induced by Ro plus light (data not shown). We modified the protocol by pre-incubating HeLa cells with 200 µM of vitamin C for 30 min, and adding the same concentration of vitamin C also during the 6 h incubation period after inducing the damage. Figure 5 shows that the repair of oxidized bases is much slower than SB rejoining, and the presence of vitamin C during the incubation period after damaging the cells still did not influence the repair of the FPG-sensitive sites induced by Ro plus light. 

### 3.5. *In Vitro* Repair Assay

**Figure 6:**
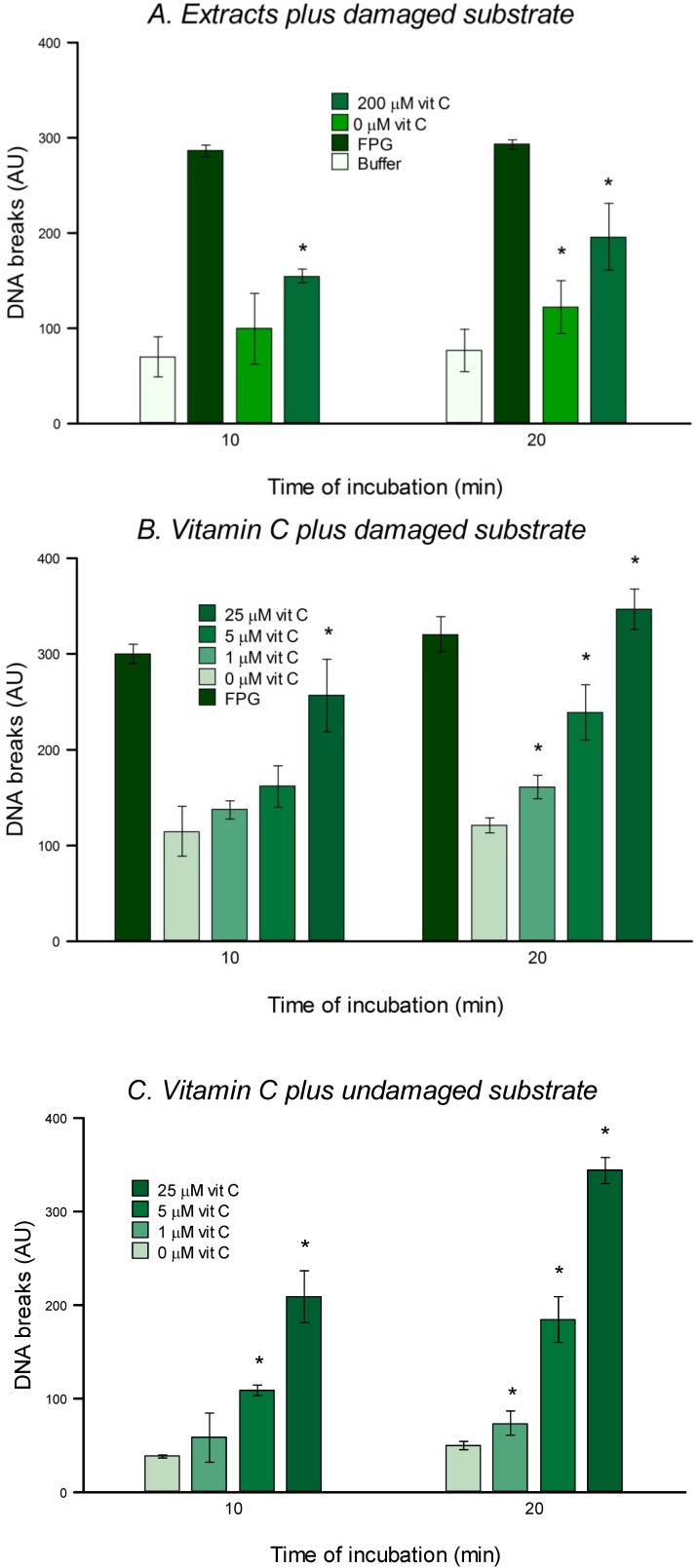
(**A**) Effect of extracts from HeLa cells pre-treated with 0 and 200 µM of vitamin C for 6 h on a DNA substrate containing oxidized purines. (**B**) Effect of different concentrations of vitamin C on a DNA substrate containing oxidized purines. (**C**) Effect of different concentrations of vitamin C on a non-damaged DNA substrate. In (**A**), Buffer F was used as a negative control. In (**A**) and (**B**), where nucleoids contained oxidized bases, FPG was employed as a positive control. Concentrations of vitamin C displayed in the box on panels (**B**) and (**C**) refer to concentrations in buffer A: final concentrations were 4× less. Bars: SD calculated from the results of 3 independent experiments. Statistical comparisons (* *p* < 0.05): vitamin C (different concentrations) *vs*. 0 μM.

As a final check as to whether vitamin C has any effect on DNA repair activity we used an *in vitro* approach, measuring the ability of extracts of vitamin C-preincubated cells to recognize and incise oxidized bases in substrate nucleoids. HeLa cells were treated with 200 µM vitamin C for 6 h. Extracts were prepared and incubated for 10 or 20 min with gel-embedded HeLa nucleoid DNA containing oxidized purines. (Previous experiments [13] have shown no significant breaks induced in undamaged substrate.) Then the comet assay was performed to measure the breaks induced by the extracts, as an indicator of repair activity. Extracts from HeLa cells preincubated with 200 μM vitamin C produced a significant increase in SBs in substrate nucleoids (compared with extracts from non-vitamin C-treated cells) at both reaction incubation times (Figure 6A). Lower concentrations of vitamin C (50 or 100 µM) did not have any effect. However, when we checked the possibility that vitamin C was having a direct effect on the DNA, we found that breaks were in fact induced in HeLa nucleoid DNA containing oxidized purines or in undamaged DNA (Figure 6B,C). A significant effect is seen at a concentration as low as 1 μM. This concentration relates to the concentration as prepared in buffer A, before 5× dilution with buffer F. Therefore the concentration capable of damaging DNA is actually as low as 0.2 μM. 

### 3.6. Uptake of Vitamin C

**Figure 7:**
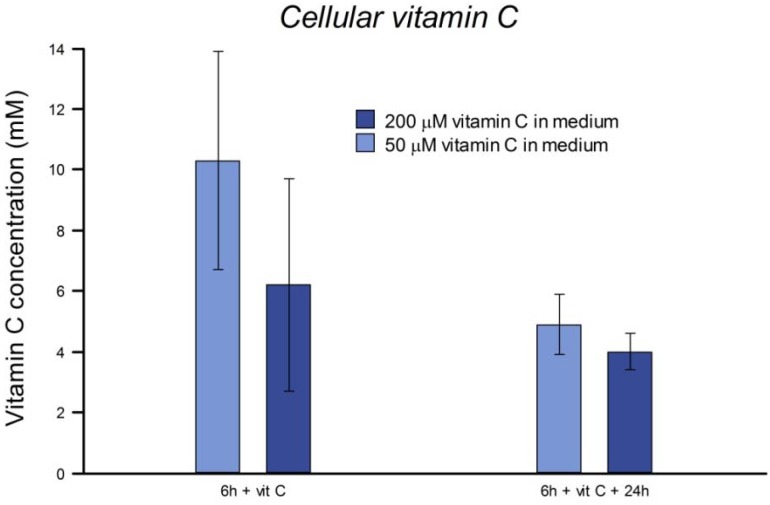
Uptake of vitamin C by HeLa cells. Cells were incubated for 6 h in medium containing vitamin C at 50 µM (light shading) or 200 µM (dark shading). Intracellular vitamin C was measured by HPLC at the end of this incubation or after a further 24 h incubation.

Concentrations of vitamin C measured in cell pellets after 6 h or 24 h incubation are shown in Figure 7. The concentration within the cells is estimated on the basis of a volume of 5 µL per 10^6^ cells. It is clear that vitamin C is taken up by HeLa cells and accumulates to a concentration far higher than that provided in the medium. 

## 4. Discussion

*In vitro*, vitamin C is capable of acting as a pro-oxidant, particularly in combination with metal ions [14], and there are concerns that it might lead to excessive DNA oxidation in humans. There are reports of increases in oxidized bases in the DNA of white blood cells after supplementation of normal subjects with vitamin C [7] or with vitamin C plus ferrous sulfate [15]. However, these studies were carried out using GC-MS to measure oxidized bases—a technique now recognized as being particularly prone to artifact and therefore unsuitable for determining low level base oxidation [16].

The saturation plasma level for vitamin C in humans is around 80 μM, and this concentration is approached with daily intake of 200 mg/day [17]—an intake that can feasibly be attained by a recommended intake of several portions of fruits and vegetables each day. The justification for exceeding in our experiments the maximum vitamin C concentration found in plasma is that vitamin C is reportedly not stable in cell culture medium; 40% depletion was detected after 2 h of incubation [18], and only about 30% remained at 6 h. 

*In vivo*, in subjects supplemented with 200 mg of vitamin C per day, active accumulation by lymphocytes results in intracellular levels saturating at 3.5 mM [17]. Welch *et al.* [19] reported a linear accumulation of vitamin C up to about 0.5 mM in 4 h in human fibroblasts incubated in 50 μM vitamin C. We found that the concentration of vitamin C inside HeLa cells after culture in medium with the vitamin was in the mM range, and was still high after 24 h. Although they have undergone many changes in their several decades of continuous culture from their origin in tumor tissue, HeLa cells evidently retain the active transport mechanisms responsible in normal cells for uptake of vitamin C [19], and so we regard them as suitable for an investigation of intracellular effects of vitamin C. 

In the present study, in spite of the high intracellular concentration of vitamin C, there was no increase in SBs or FPG-sensitive sites. Others have reported no increase in SBs and/or oxidized bases in various cell types after incubation with vitamin C—in human HepG2 liver-derived cells with 10 μM vitamin C [20], in primary human fibroblasts incubated with 100 μM vitamin C [21], and most recently in HL-60 cells (human leukemic cell line) incubated with concentrations of 50–250 μM vitamin C [22,23].

In our experiments, preincubation with up to 200 μM vitamin C had no significant effect on the level of H_2_O_2_-induced breaks or 8-oxoGua induced by treatment with Ro plus light. There are mixed reports on the effect of pre-incubation with vitamin C on the DNA damage inflicted by genotoxic agents. Vitamin C pre-incubation (30 min, 10 or 50 μM) decreased the SBs caused by streptozotocin in lymphocytes or HeLa cells [24], and protected against the effect of NiCl_2_ in lymphocytes [25]. Duarte and Jones [21] preincubated primary human fibroblasts for 12 h with 20, 100 or 500 μM vitamin C, and—also using the comet assay—found an increase in H_2_O_2_-induced damage. Carbofuran-induced damage to human lymphocyte DNA was decreased by preincubation with 30 μM vitamin C [26]. Similar protection was afforded by 10–50 μM vitamin C pretreatment against damage induced by 2-hydroxyethyl methacrylate and urethane dimethacrylate (used in dental treatment) [27,28].

When vitamin C is present simultaneously with a genotoxic agent (rather than during a preincubation), potentiation of the damaging effect of the latter may be seen—even when the effect of preincubation is a protective one. Such a dual effect was reported by Wozniak and Blasiak [25] for NiCl_2_. Vanadyl sulfate led to more DNA breaks in HeLa cells and lymphocytes when they were co-incubated with vitamin C for 1 h [29], perhaps as a result of a metal ion-vitamin C interaction. Both DNA breaks and oxidized purines were induced when HL-60 cells were incubated with vitamin C and copper (II) sulfate, but no such damage was caused by vitamin C alone [23]. Vitamin C was unable to protect human submandibular gland and oral epithelial cells against DNA damage induced by bracken fern, and in fact showed a synergistic effect on DNA breakage [30]. In contrast, co-incubation of HepG2 cells with both vitamin C and *N*-nitrosamines resulted in fewer DNA breaks/oxidized bases [20]—perhaps an effect of vitamin C mediated through xenobiotic metabolizing enzymes. 

To summarize our results relating to DNA damage, in HeLa cells—which clearly are able to take up and accumulate vitamin C—we find no sign of either pro- or anti-oxidant effects.

There are some human intervention studies that suggest a possible regulatory role for vitamin C in the repair of oxidized DNA—a topic highlighted by Sram *et al.* in their recent review [8] as deserving further attention. Cooke *et al*. [31] measured 8-oxodGuo in lymphocyte DNA, serum and urine from 30 healthy volunteers involved in a 6 week placebo and 6 week vitamin C (500 mg/day) supplementation study (plus a washout period). They found a decrease in the levels of 8-oxodGuo in DNA and an increase in urine and serum. In addition to reservations about the less than ideal study design and the likely errors in the measurement of 8-oxodGuo, the interpretation of the results is questionable, since the presence of 8-oxodGuo in serum or urine does not directly reflect OGG activity; the base, not the nucleoside, is excised during BER. Tarng *et al.* [32] supplemented chronic hemodialysis patients with 300 mg of vitamin C, and found a significant upregulation of *hOGG1* mRNA at 24 h after vitamin C administration. Astley *et al.* [33] took lymphocytes from human volunteers after 3 weeks of supplementation with the modest dose of 60 mg vitamin C per 2 days. There was no significant effect on plasma vitamin C, and no change in repair capacity (detected as repair synthesis on an oxidatively damaged plasmid template). 

Supplementation for 3 weeks with green kiwifruits led to a significant increase in plasma vitamin C, decreases in endogenous base oxidation and H_2_O_2_-induced damage *ex vivo*, and an enhancement of lymphocyte BER on a substrate containing 8-oxoGua [34]; however, golden kiwifruits modestly increased plasma vitamin C and showed protection against DNA oxidation but no effect on DNA repair [35]. Guarnieri *et al.* [36] supplemented volunteers with 0.5 g/day vitamin C in normal or slow release form (together with vitamin E); only the slow release form stimulated BER capacity in lymphocytes. 

Reports in the literature on effects of vitamin C on DNA repair in cells in culture are sparse. Konopacka *et al.* [37] irradiated mouse lymphocytes with 2 Gy of γ-rays, and then incubated them with or without vitamin C, vitamin E and β-carotene; rejoining of strand breaks was faster in the presence of antioxidants.

We report here that vitamin C has no effect on cellular repair of SBs or oxidized bases in HeLa cells. The *in vitro* test for 8-oxoGua DNA glycosylase (OGG) activity could not be carried out, since it was evident that vitamin C itself can cause damage to nucleoid DNA (Figure 6), presumably acting as a pro-oxidant (as mentioned in the Introduction). This cleavage could be mediated via production of hydroxyl radicals in the presence of DNA-bound transition metal such as copper [14].

The reason for native nuclear DNA being resistant to attack by vitamin C, while nucleoid DNA is susceptible, is not clear, though it may be that this apparently pro-oxidant effect of vitamin C is prevented by the high concentrations of the thiol-rich antioxidant glutathione present in the intact nucleus. Bergstrom *et al.* [22,23] report the oxidation of dGuo *in vitro* by vitamin C. To summarize, we have found no evidence in HeLa cells for pro-oxidant effects of vitamin C at extracellular concentrations up to 200 μM, but also no evidence for antioxidant protection. Nor did we see any effect on the repair of SBs or oxidized bases. Vitamin C at low concentration is, however, capable of damaging nucleoid DNA. The same strategy was used in our laboratory to study the antioxidant/pro-oxidant effects of β-cryptoxanthin [13]. Our results showed that this carotenoid does not cause DNA damage but, in contrast to vitamin C, it protects HeLa cells from damage induced by H_2_O_2_ or photosensitizer plus light. In addition, it enhances DNA repair, as measured with the same assays as were used in the present report.
